# Occurrence of zoonotic parasites in economically important fish species from the Northeastern region of Brazil

**DOI:** 10.1590/S1984-29612026034

**Published:** 2026-08-03

**Authors:** Noemi de Castro Torres, Brenda Madruga Rosa, Sarah Leticia Paiva Oliveira, Bruna Mesquita de Moraes, Beatriz Menezes Lins, Rodney Kozlowiski de Azevedo, Vanessa Doro Abdallah

**Affiliations:** 1 Universidade Federal de Alagoas – UFAL, Instituto de Ciências Biológicas e da Saúde – ICBS, Setor de Parasitologia e Patologia, Maceió, AL, Brasil; 2 Universidade Federal de Alagoas – UFAL, Campus de Engenharia e Ciências Agrárias – CECA, Programa de Pós-graduação em Ciência Animal – PPGCA, Rio Largo, AL, Brasil

**Keywords:** Public health, fish consumption, nematodes, Saúde pública, consumo de peixe, nematódeos

## Abstract

This study aimed to identify and analyze zoonotic parasites found in *Scomberomorus cavalla* and *Trichiurus lepturus* collected from the Mundaú-Manguaba Estuarine Lagoon Complex (CELMM), considering their commercial relevance and the associated risks to food safety and public health. A total of 34 fish were obtained from local fishermen, including 30 *T. lepturus* and four *S. cavalla*. The fish were necropsied and their internal organs were examined to identify parasites. Prevalence, abundance, and mean intensity of parasitic infections were calculated. In *T. lepturus*, the most prevalent (20%) nematode was *Contracaecum* sp., predominantly found in the coelomic cavity. In *S. cavalla*, the most frequently observed one (100% prevalence) was *Hysterothylacium* sp., with infections disseminated in the stomach, intestine, liver, and coelomic cavity. All identified parasites were documented for the first time in fish collected from this estuarine complex, CELMM, in Brazil, expanding their known geographical and ecological distribution. The presence of zoonotic parasites in commercially sold fish from CELMM poses a significant public health risk. Therefore, stringent sanitary inspection measures and public awareness initiatives are essential to mitigate contamination risks and ensure the quality of fish products intended for human consumption.

## Resumo

Este estudo teve como objetivo identificar e analisar parasitos zoonóticos encontrados em *Scomberomorus cavalla* e *Trichiurus lepturus* coletados do Complexo Estuarino Lagunar Mundaú-Manguaba (CELMM), considerando sua relevância comercial e os riscos associados à segurança alimentar e saúde pública. Um total de 34 peixes foram obtidos de pescadores locais, incluindo 30 *Trichiurus lepturus* e quatro *Scomberomorus cavalla*. Os peixes foram necropsiados e seus órgãos internos foram examinados à procura de parasitos. Prevalência, abundância média e intensidade média da infecção parasitária foram calculadas. Em *T. lepturus*, o nematódeo com maior prevalência (20%) foi *Contracaecum* sp., predominantemente encontrado na cavidade celômica. Em *S. cavalla*, *Hysterothylacium* sp. obteve a maior prevalência (100%), com infecção disseminada pelo estômago, intestino, fígado e cavidade celômica. Todos os parasitos identificados foram documentados pela primeira vez em peixes coletados nesse complexo estuarino, CELMM, do Brasil, expandindo sua distribuição geográfica e ecológica. A presença de parasitos zoonóticos em peixes comercializados vindos do CELMM impõe um risco significativo à saúde pública. Portanto, medidas para inspeções sanitárias rigorosas e iniciativas de conscientização pública são essenciais para mitigar riscos de contaminação e garantir a qualidade do pescado pretendido para consumo humano.

## Introduction

Although often overlooked in studies on biodiversity and ecology, fish parasites are essential components of biodiversity (Sures, 2008). The occurrence of zoonotic parasites in fish raises concerns about food safety, as these organisms can cause severe diseases, leading to significant public health issues (Cardia & Bresciani, 2012). The risk of contamination increases when these parasites migrate into the fish musculature (Okumura et al., 1999). Given the growing preference among Brazilians for social and cultural food habits inspired by oriental cuisine, the demand for raw fish dishes such as sushi and sashimi has increased, presenting new challenges for food safety and public health (Cruz et al., 2010; Gomes et al., 2020).

Nematode parasites from the families Anisakidae and Raphidascarididae are frequently found in marine fish, and humans may become accidental hosts through the consumption of raw or undercooked contaminated fish (Nonković et al., 2025). Parasitic infections associated with anisakids have become a major focus of emerging research in foodborne disease risk and public health, with various countries dedicating efforts to understanding host-parasite dynamics and associated infection processes (Lam et al., 2024).

*Scomberomorus cavalla* (Cuvier, 1829), commonly known as king mackerel, has a broad geographical distribution, occurring along the eastern coast of the Americas from Massachusetts, USA, to southern Brazil (Figueiredo & Menezes, 2000). In Brazil, the species' commercial importance is reflected in consumption data (Dias et al., 2011).

Similarly, *Trichiurus lepturus* (Linnaeus, 1758), popularly called largehead hairtail, is found in tropical and temperate waters worldwide (Menezes et al., 2003). It ranks among the top six most heavily fished species globally (Bittar et al., 2008). Both *S. cavalla* and *T. lepturus* are of considerable importance not only due to their commercial appeal and high fishing volume but also because of the public health and food safety challenges they pose. Additionally, in both host species analyzed in this study, the presence of zoonotic potential nematodes has already been recorded and in many regions of the world, in studies such as Klein (1973), Oliveira da Silva et al. (2000a), Dias et al. (2011) and Youssir et al. (2017).

Despite the strong interconnection between aquatic ecosystems, health, economy, and parasitism, fish parasites are not as researched as those of other hosts in the field of parasitology. As both of the fish species here analyzed for parasites are economically significant, this study aimed to search for and identify zoonotic parasites found in the *S. cavalla* and *T. lepturus* collected from the Mundaú-Manguaba Estuarine Lagoon Complex (CELMM), specially being an environment that holds significant local commercial importance.

## Material and Methods

### Study area

The Mundaú-Manguaba Estuarine Lagoon Complex (CELMM) consists of the Mundaú Lagoon, covering approximately 23 km^2^. Together with the Manguaba Lagoon, it forms a complex with a total area of approximately 55 km^2^ (Tamano et al., 2015). The Complex stands out not only for its habitat diversity but also for its abundant fishery resources, which include various fish and macrocrustacean species (Tamano et al., 2015). Located in the state of Alagoas, Brazil, the complex holds both ecological and socio-economic significance. Ecologically, it provides a vital habitat for a rich biodiversity, featuring freshwater areas, mangroves, brackish waters, restinga vegetation, and beaches, which support a complex food web and serve as a nursery for numerous marine species (Arceo-Carranza et al., 2021). From an economic perspective, CELMM sustains fisheries that are crucial for local livelihoods and the regional economy (Salles, 1995).

### Fish analysis

The fish used in this study were purchased from local fishermen in the vicinity of CELMM between April 2022 and April 2023. Ethical approval from the Animal Use Ethics Committee (CEUA) was not required, as the fish were already dead when acquired. The total length of each fish was measured and each one was compared to the length at first maturity, to confirm that the fish had reached sexual maturity, according to fishbase (Froese & Pauly, 2026). A total of 34 fish specimens were necropsied, including 30 *T. lepturus* and four *S. cavalla*. The intestines, liver, gonads, and stomach were removed and agitated with water, so the existing parasites would loosen to facilitate the collection. The coelomic cavity was also washed with water to search for parasites in transit from the internal organs to the muscle. The material was then filtered using a 75 μm sieve and examined under a stereoscopic microscope using individual Petri dishes (Eiras et al., 2000). The muscular tissue of the fish was not filleted nor analyzed.

### Parasite analysis

Parasite preparing techniques followed established protocols, as they were stored in 70% ethanol, then put on lactophenol for clarification (Eiras et al., 2000). For morphological analysis at the generic level, the specimens were then observed under a Nikon Eclipse E200 microscope, where morphometric analysis was conducted. Images were captured using an Opticam OPZTS camera with 5.3 megapixels and an Opticam 0400S microscope with a 10.5-megapixel camera. The taxonomic classification and morphological terminology for anisakid and raphidascaridid larvae followed dichotomous keys by Tavares & Luque (2006) and prevalence, average abundance and average intensity were calculated according to Bush et al. (1997).

### Mapping of parasite registry

Occurrence maps for parasites (Anisakidae and Raphidascaridiidae) found infecting the fish species analyzed in this study were developed using the software QGIS, with the WGS84 datum (EPSG 7030) (QGIS, 2025) and the coordinates for previous registers from around the world were obtained from the studies mentioned in Tables 1 and 2.

**Table 1:** Records of parasitic infections by nematodes with zoonotic potential in *T. lepturus* from different locations.

**Parasites**	**Prevalence (%)**	**Average intensity**	**Average abundance**	**Site of infection**	**Local**
*Anisakis*sp.	-	-	-	Mesentery	Rio de Janeiro (Oliveira da Silva et al., 2000a)
*Anisakis* sp.	12.70	1.10±0.30	0.10±0.40	-	Rio de Janeiro (Oliveira da Silva et al., 2000b)
*Anisakis* sp.	88.30	171.12± 343.40	151.20± 327.30	Mesentery	Rio de Janeiro (Carvalho & Luque, 2011)
*Anisakis* sp.	45.83	-	-	Oral cavity, mesentery, gastrointestinal tract, liver and gonads	Indonesia (Setyobudi et al., 2011)
*Anisakis*sp.	3.00	1.00	0.03	Gonads	Alagoas *
*Anisakis typica*	20.31	-	-	Cavity and mesentery	Rio de Janeiro (Borges et al., 2012)
*Anisakis simplex*	59.04/ 10.95	-	-	Cavity/ Musculature	Morocco (Youssir et al., 2017)
*Contracaecum*sp.	-	-	-	Mesentery	Rio de Janeiro (Oliveira da Silva et al., 2000a)
*Contracaecum*sp.	91	67.60±73.30	61.40±72.50	-	Rio de Janeiro (Oliveira da Silva et al., 2000b)
*Contracaecum* sp.	20.00	1.57	0.36	Stomach/ Cavity/ Gonads	Alagoas *
*Contracaecum verrucosum*	100.00	213.67	213.67	Integument, gills and stomach	Rio Grande do Norte (Cavalcanti et al., 2012)
*Hysterothylacium* sp.	-	-	-	Mesentery	Rio de Janeiro (Oliveira da Silva et al., 2000a)
*Hysterothylacium* sp.	3.60	1.50±0.70	<0.10	-	Rio de Janeiro (Oliveira da Silva et al., 2000b)
*Hysterothylacium* sp.	51.56	-	-	Cavity and mesentery	Rio de Janeiro (Borges et al., 2012)
*Hysterothylacium*sp.	33.33	1	0.33	Intestine	Rio Grande do Norte (Cavalcanti et al., 2012)
*Hysterothylacium*sp.	3.00	1.00	0.03	Cavity	Alagoas *
*Raphidascaris*sp.	-	-	-	Mesentery	Rio de Janeiro (Oliveira da Silva et al., 2000a)
*Raphidascaris*sp.	23.60	2±2.20	0.50±1.30	-	Rio de Janeiro (Oliveira da Silva et al., 2000b)
*Raphidascaris*sp.	6.00	1.00	0.06	Cavity/ Stomach	Alagoas^*^

* Present study.

**Table 2:** Records of parasitic infections by nematodes with zoonotic potential in *S. cavalla* from different locations.

**Parasites**	**Prevalence (%)**	**Average intensity**	**Average abundance**	**Site of infection**	**Local**
*Anisakis*sp.	1.00	2.0	0.02	Serosa of the stomach	Rio de Janeiro (Dias et al., 2011)
*Contracaecum*sp.	16.0	3.25	0.86	Mesentery	Rio de Janeiro (Dias et al., 2011)
*Hysterothylacium fortalezae*	-	-	-	Stomach, small intestine and large intestine	Ceará (Klein, 1973)
*Hysterothylacium* sp.	100.00	3.81	3.81	Stomach/ Intestine /Liver	Alagoas^*^
*Raphidascaris*sp.	25.00	2.50	0.93	Cavity	Alagoas^*^
*Terranova trichiuri*	-	-	-	Stomach and gonads	Ceará (Klein, 1973)
*Terranova*sp.	50.00	4.00	2.50	Stomach/ Intestine/ Liver/ Cavity	Alagoas^*^

* Present study.

## Results

The total length of *T. lepturus* averaged 57.69 ± 6.36 cm, while *S. cavalla* measured 49.625 ± 3.56 cm, meaning all the collected specimens, from both species, had already reached maturity, although were considered to be on the younger side. Of the 34 fish necropsied, 16 out of 30 *T. lepturus* specimens and all four *S. cavalla* specimens were infected with zoonotic nematodes.

Five nematode genera were found, all in stages L3 or L4: *Anisakis* (Figure 1), *Contracaecum* (Figure 1), *Terranova* (Figure 1) (Anisakidae)*, Hysterothylacium* (Figure 2) and *Raphidascaris* (Figure 2) (Raphidascaridiidae). The first two were found only in *T. lepturus* and *Terranova* sp., only in *S. cavalla*. Although *Terranova* sp. is now a taxon inquirendum, we have decided to keep the previous classification, due to our specimens not fitting the description keys from both new genera (*Euterranova* sp. and *Neoterranova* sp. Moravec & Justine, 2020) (Moravec & Justine, 2020).

**Figure 1:**
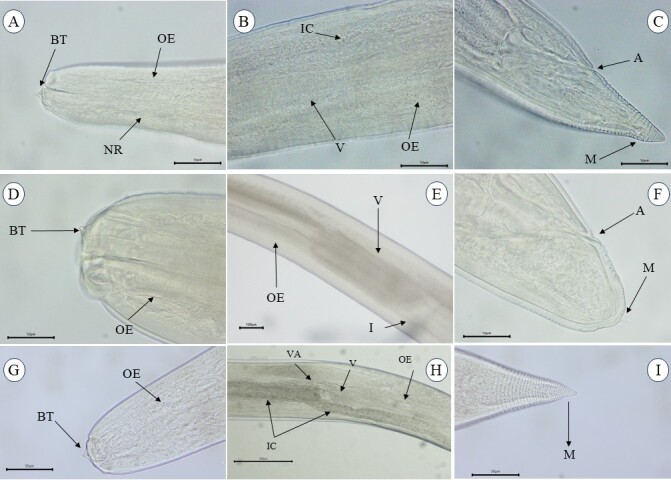
Nematodes from the Anisakidae family. A-C:*Terranova*sp. A-Showing the anterior end, with boring tooth (BT), oesophagus (OE) and nerve ring (NR). B- Ventriculus (V), intestinal caecum (IC) and oesophagus (OE). C- Posterior end, with the anus (A) and lack of mucron (M). D-E:*Anisakis*sp. D- Anterior end in 40x magnification, presenting boring tooth (BT) and oesophagus (OE). E- Midsection of the parasite showing the oesophagus (OE), ventriculus (V) and intestine (I). F- Posterior end, with mucron (M) and anus (A). G-I:*Contracaecum*sp. G- Anterior end, showing boring tooth (BT) and oesophagus (OE). H- Midsection of the body, with intestinal caecum (IC), oesophagus (OE), ventriculus (V) and the ventricular appendix (VA). I- Posterior end showing the lack of mucron (M).

**Figure 2:**
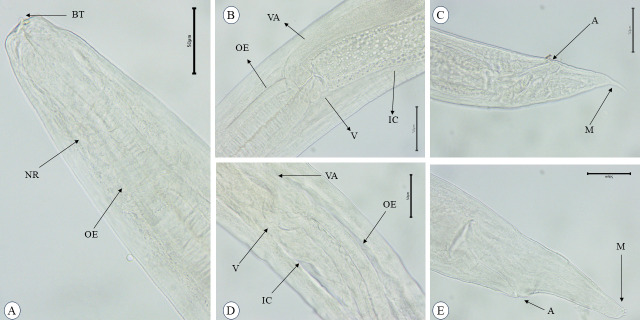
Nematodes from Raphidascaridiidae. A-C:*Raphidascaris*sp. A- Anterior end, showing boring tooth (BT), nerve ring (NR) and oesophagus (OE). B- Midsection, showing the oesophagus, ventriculus (V), ventricular appendix (VA) and the intestinal caecum (IC). C- Posterior end, showing anus (A) and mucron (M). D-E:*Hysterothylacium*sp. D- Midsection with oesophagus (OE), intestinal caecum (IC), ventriculus (V) and ventricular appendix (VA). E- posterior end showing anus (A) and mucron (M).

In *T. lepturus*, the most prevalent nematode was *Contracaecum* sp. (20%), with the coelomic cavity being the most common site of infection (Table 1). Conversely, in *S. cavalla*, *Hysterothylacium* sp. was the most prevalent nematode (100%), with infection sites occurring similarly in the stomach, intestine, liver, and coelomic cavity (Table 2).

## Discussion

Overall, the present study revealed distinct patterns of nematode infection between the host species analyzed, while also highlighting novel host–parasite associations and expanding the known geographic distribution of these taxa along the Alagoas coast. Infection by the nematodes in *T. lepturus* was predominantly caused by larvae of the genus *Contracaecum*, a parasite previously reported at high prevalence in other locations for this host species (Oliveira da Silva et al., 2000a, b) (Table 1). Meanwhile, *S. cavalla* exhibited the highest prevalence of *Hysterothylacium* sp. (Table 2), marking the first recorded occurrence of this genus in this host species.

All parasites identified in this study are being documented for the first time parasitizing fish collected in this location, thereby expanding their known geographic distribution (Figure 3), (keeping in mind the migration pattern of these fish species and their estimate age) especially when Alagoas is only the third world location to have registered such parasites in *S. cavalla* and the fourth Brazilian state for *T. lepturus*, enhancing the importance of this study.

**Figure 3:**
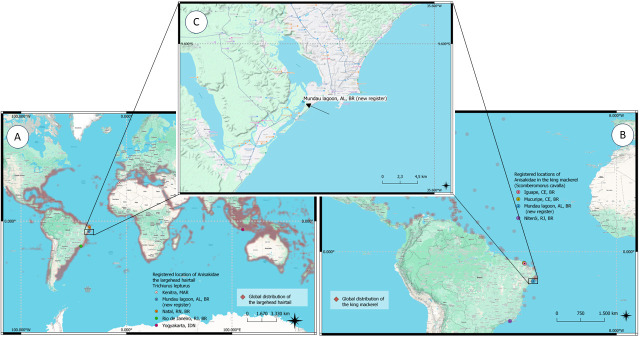
Maps of the global distribution of *Trichiurus lepturus* (A) and *Scomberomorus cavalla* (B) with the new locality register of occurrence of Anisakidae (Mundaú Lagoon, Alagoas, Brazil) (C)*.*

The prevalence, average abundance and average intensity of the parasites from *T. lepturus* (Table 1) found in this study were low compared to the previous studies done on this species. That could be explained by the age of the fish, since, for example, in Oliveira da Silva et al. (2000b), the fish averaged 122.7 ± 8.6 (108-148) cm, which is double the size of the ones analyzed in this study. Therefore, it should be taken into account that the feeding habits are slightly different and that could alter the parasitic load in the fish (Nakamura & Parin, 1993). As for *S. cavalla* (Table 2), considering the parasites solely as nematodes and compared to the study conducted by Dias et al. (2011), the fish in this study did present higher prevalence of the nematodes, however if only each genera is taken into consideration, we cannot compare the results, since the parasites found are from different genera.

Anisakidosis and anisakiasis result from the ingestion of infective larvae from the family Anisakidae, with *A. simplex, Pseudoterranova decipiens*, and *C. osculatum* being the most relevant species in public health (Souza et al., 2016). This zoonotic disease occurs frequently due to the broad diversity of fish species affected by these parasites (Santos et al., 2020). In this study, *Anisakis* and *Contracaecum* species were reported parasitizing *T. lepturus*, a species commonly used raw in Japanese-like cuisine, reinforcing the significance of this study.

In both species studied, parasites were found in the coelomic cavity and internal organs. While their presence in the mesentery poses a lower zoonotic risk, there remains the possibility of larval migration into fish musculature after capture and freezing (Alves et al., 2020). Most of the studies done in parasitology for these fish species have not registered the presence of these zoonotic nematodes in the muscle, except for Youssir et al. (2017), however even then, the prevalence was low and as appointed by the authors, the larvae was “rarely observed in the musculature”.

The life cycle of the parasites should also have a substantial weight when looking at sites of infection, since the nematodes found in this research were larvae (L3 or L4) (Figures 1 and 2) and therefore the fish examined were not their definitive host and within these intermediate hosts their preferred location is the gastrointestinal tract, as seen in the studies referenced in this paper (Klein, 1973; Oliveira da Silva et al., 2000a, b; Carvalho & Luque, 2011; Setyobudi et al., 2011; Cavalcanti et al., 2012; Borges et al., 2012; Dias et al., 2011; Youssir et al., 2017)

Food safety is a fundamental issue, however it is not as closely monitored as it should be, since it was possible to buy the fish and find parasites with the potential to infect humans, although Brazil has a set of laws and guidelines that in theory protects the population from that exactly. Article 6, Paragraph 1 of Law No. 8.080/90 highlights the importance of sanitary surveillance, under the responsibility of the National Health Surveillance Agency (Anvisa), aimed at preventing foodborne diseases from consuming unsafe products (Brasil, 1990). To ensure safety, Decree No. 9.013 of March 29, 2017, establishes strict guidelines for the control and treatment of animal-derived products. Article 212 reinforces parasite control according to the regulations of the Ministry of Agriculture, Livestock, and Supply (MAPA) (Brasil, 2017), while Article 214 allows for the conditional use of products, provided there is no health risk. Article 215 recommends methods such as freezing, salting, and heating to reduce or eliminate parasites in food, and Article 499 determines that products with extensive muscular parasite infections are unsuitable for consumption (Neto, 2020).

Strict inspections and sanitary control are essential to ensure food safety in fish products (Seta et al., 2017). Due to the high contamination risk in their natural environment, frequent exposure, and processing conditions, fish are highly susceptible to bacterial and parasitic contamination (Paiva et al., 2018). In this scenario, fish and their derivatives, commonly consumed by the population, may harbor parasites that pose health risks when ingested accidentally (Silva et al., 2008).

In Brazil, especially in the Northeast, fish consumption is significantly high, with an annual per capita average of 15 kg in the region and 9 kg nationwide, where fishing is a key economic activity (Brasil, 2017). Literature reports indicate cases where consuming cooked fish may not completely eliminate health risks, as certain parasite proteins are heat-resistant and can trigger anaphylactic reactions (Bao et al., 2019).

A substantial increase in anisakid infections worldwide has been observed over the past three decades (Silva et al., 2008). A systematic literature review provided recent data estimating a global incidence rate of 0.32 per 1,000,000 inhabitants (Orphanet, 2024). Japan stands out as the country with the highest estimated anisakiasis cases (Ohnishi et al., 2023). However, with the growing detection of *Anisakis* larvae in the country, Japanese consumers have become increasingly cautious about food safety (Hochberg & Hamer, 2010).

The consumption of raw fish in Brazil, including species such as *S. cavalla* and *T. lepturus*, has been rising in popularity. These fish are highly accepted commercially and may be consumed in both oriental cuisine practices and improperly prepared dishes (Masson & Pinto, 1998).

As the muscular tissue was not fully analyzed, it is understandable that the quantitative methods in this study might not be completely accurate, however that doesn’t take away the risk of infection by eating these fish if parasitized, especially as the abundance may have been underestimated and adding to that, as mentioned previously, only one of all the studies done on Anisakidae parasitizing these fish species did register their presence in the muscle (Youssir et al., 2017).

Considering these facts and the increasing consumption of raw fish in Brazil, proper preparation and inspection practices are crucial in reducing the incidence of parasitic infections.

## Conclusion

Brazil has various regions with high fish consumption. This study highlights the need for increasing inspection, specially of fish that are sold fresh. These findings are essential for assessing the real risks associated with fish consumption in Brazil, even more so with the increase in consumption of raw or undercooked fish. More studies like this one can help with defining sanitary public policies and ensure food safety.

## Data Availability

The data used in this paper will be available to the public.
